# Detecting Semantic Priming at the Single-Trial Level

**DOI:** 10.1371/journal.pone.0060377

**Published:** 2013-04-02

**Authors:** Jeroen Geuze, Marcel A. J. van Gerven, Jason Farquhar, Peter Desain

**Affiliations:** Radboud University Nijmegen, Donders Institute for Brain, Cognition, and Behaviour, Nijmegen, The Netherlands; University Of Cambridge, United Kingdom

## Abstract

Semantic priming is usually studied by examining ERPs over many trials and subjects. This article aims at detecting semantic priming at the single-trial level. By using machine learning techniques it is possible to analyse and classify short traces of brain activity, which could, for example, be used to build a Brain Computer Interface (BCI). This article describes an experiment where subjects were presented with word pairs and asked to decide whether the words were related or not. A classifier was trained to determine whether the subjects judged words as related or unrelated based on one second of EEG data. The results show that the classifier accuracy when training per subject varies between 54% and 67%, and is significantly above chance level for all subjects (N  = 12) and the accuracy when training over subjects varies between 51% and 63%, and is significantly above chance level for 11 subjects, pointing to a general effect.

## Introduction

Semantic priming with written word pairs has been investigated since the first study by Mayer and Schwaneveldt [1]. In this first experiment subjects were asked to indicate whether pairs of strings were in the same or in a different category, where the categories were words and non-words. The first string in the pair is called the *prime* and the second is called the *probe*. When both prime and probe were words they could either be related or unrelated. The authors showed that there was a difference in response times and errors made when both strings were related words versus when they were unrelated words.

However, Meyer and Schwaneveldt [1] only studied behavioral effects. Kutas and Hillyard [2] published the first semantic priming experiment where they also investigated brain potentials. They studied the N400 ERP component, a negative going wave around 400 ms after word onset, in the response to sentence-final words. They presented sentences which ended in an expected word, a word related to the expected word, or a word unrelated to the expected word. The response to a word expected based on the sentence context resulted in the smallest N400 peak. Words that were unrelated to the expected word resulted in the largest N400 peak. Words that were related to the expected word showed a N400 peak amplitude that was between the expected and related word responses. Where Kutas and Hillyard [2] showed this effect for words in a sentence, Rugg [3] and Bentin et al. [4] showed this effect also occurs with words in isolation.

A number of theories and models have been developed to explain this phenomenon, i.e., the spreading activation model [5], the compound-cue retrieval theory [6], and the distributed memory model [7]. The spreading activation model is based on the assumption that activation spreads from one node (the prime-word) to surrounding nodes (related words) which facilitates retrieval of related probes as their nodes are already activated. In the compound-cue retrieval theory, prime and probe are combined to form the compound cue, which is used to access memory. If the compounds are associated in memory it facilitates responses to the probe. The distributed memory model states that words are not single nodes, but consist of a distributed collection of nodes representing their characteristics. When some of these characteristics are activated by a related prime-word, it facilitates responses to probe-words. All three models have in common that they model the automatic process of lexical access. There is a long-standing debate on whether priming is only influenced by automatic processes (lexical access) or is also influenced by controlled processes (lexical integration) [8]–[10], and which of these processes is the basis of the N400 effect found in semantic priming studies. Although evidence has been gathered for both theories, there is no conclusive answer yet. Providing evidence for one of the above-mentioned theories falls outside the scope of this article.

The studies mentioned above only examine grand average ERPs, where for each condition several hundred examples are averaged, requiring hours of measurement time spread over multiple subjects. However, machine learning techniques [11] have successfully been applied to detect differences in brain responses between conditions at the single-trial level [12], requiring just seconds to minutes of measurement time with a single subject. This means that, after a short training period, an algorithm is able to determine whether a short period of EEG data is the response to one condition or the other. The P300 brain component, elicited by an odd-ball paradigm is an example of an ERP that can be successfully detected at the single-trial level [13], [14]. A brain-computer interface (BCI) is an example of an application of single-trial level detection of ERP components. A BCI allows subjects or patients to control a device, usually a computer, based exclusively on brain activity [12]. The current article aims at determining whether similar success can be achieved by using the N400 component as elicited by a semantic priming experiment.

Van Vliet, Mühl, Reuderink, and Poel [15] showed that semantic priming not only occurs when the subject is explicitly primed with a word or picture, but also when subjects prime themselves by thinking of a certain word or object. If subjects are able to prime themselves and it is possible to accurately detect priming on the single-trial level, it may be feasible to predict which concept a subject is thinking of.

In this work we want to answer the following basic question: *‘Is it possible to reliably detect semantic priming at the single-trial level?’* Our hypothesis is that semantic priming is detectable at the single-trial level and that accuracy differs significantly from chance level. It is established that the N400 amplitude is correlated with the degree of association or relatedness [16]. However, as this is a first study we chose to focus on distinguishing between strongly related and unrelated word pairs. The relatedness is determined by using the Leuven association database [17]. For the related word pairs we tried to select the word pairs with the highest association strength, without resorting to the use of synonyms.

## Methods

### Ethics Statement

The procedures used in the experiment were according the Declaration of Helsinki, and all subjects gave written informed consent. The procedures were approved by the Ethical Committee of the Faculty of Social Sciences at the Radboud University Nijmegen.

### Subjects

Measurements were obtained from 12 native Dutch subjects, 7 of whom were female. They were aged between 22 and 33 with a mean of 26.75 (±3.08). All subjects had normal or corrected-to-normal vision and were free of medication and without central nervous system abnormalities. Subjects participated in the study voluntarily, signed an informed consent form, and did not receive a reward.

### Stimuli

The stimuli consisted of two sets of Dutch word pairs: related and unrelated word pairs. The superset of related words was constructed by choosing 400 word pairs from the Leuven association dataset [17]. The Leuven association dataset was constructed by having subjects perform a continuous word association task. The cues were constructed by the researchers, while the associated words were generated by the subjects. For each word pair their association strength was determined by dividing the number of times the response was given to that particular cue by the total number of responses to that cue. 400 pairs were selected for which the association strength exceeded 0.1, i.e., word pairs where that word was given in more than 10% of the responses.

The superset of unrelated words was constructed by combining 400 cue words from the Leuven association dataset with random word forms obtained from the Celex database [18], making sure the random combination did not already occur in the Leuven association dataset.

Both sets were constructed in such a way that all 1600 words were unique. In the current experiment, the cues, constructed by the researchers of the Leuven dataset, were used as primes and the responses given by the subjects were used as probes.

To exclude confounding factors the stimuli in the two conditions were matched for word occurrence, number of letters and number of syllables. A matching program [19] was used to select 200 pairs from each of the two supersets in such a way that both primes and probes were matched for the confounding factors. The results of the matching are shown in Table 1. A number of example stimuli can be found in Table 2. A full list of stimuli can be found in the supporting information: *Stimuli S1*.

**Table 1:** Stimulus matching properties for the related and unrelated sets.

Property	Min	Max	Mean	STD
Unrelated				
Prime LogFreq	0	2.48	0.55	0.60
Probe LogFreq	0	2.76	0.78	0.61
Prime LettCnt	3	16	6.50	2.66
Probe LettCnt	3	12	5.94	1.99
Prime SylCnt	1	5	2.07	0.95
Probe SylCnt	1	4	1.82	0.76
Related				
Prime LogFreq	0	2.46	0.55	0.60
Probe LogFreq	0	2.63	0.72	0.62
Prime LettCnt	3	16	6.64	2.51
Probe LettCnt	3	12	6.45	2.22
Prime SylCnt	1	6	2.08	0.93
Probe SylCnt	1	4	1.95	0.77

LogFreq: Logarithm of word frequency, LettCnt: Number of letters, SylCnt: Number of syllables.

**Table 2:** Examples of stimuli used in the experiment.

Prime		Probe
Unrelated		
tang (pliers)	–	opbrengst (yield)
berg (mountain)	–	drankje (small drink)
eland (moose)	–	eerbied (respect)
rog (ray)	–	maaier (mower)
inktvis (squid)	–	tentakel (tentacle)
slurf (trunk)	–	olifant (elephant)
Related		
mier (ant)	–	klein (small)
tram (tram)	–	spoor (track)
racket (racket)	–	tennis (tennis)
naald (needle)	–	draad (thread)
gesp (buckle)	–	reflectie (reflection)
specht (woodpecker)	–	verpleger (male nurse)

Taken from the related and unrelated sets.

**Figure 1:**
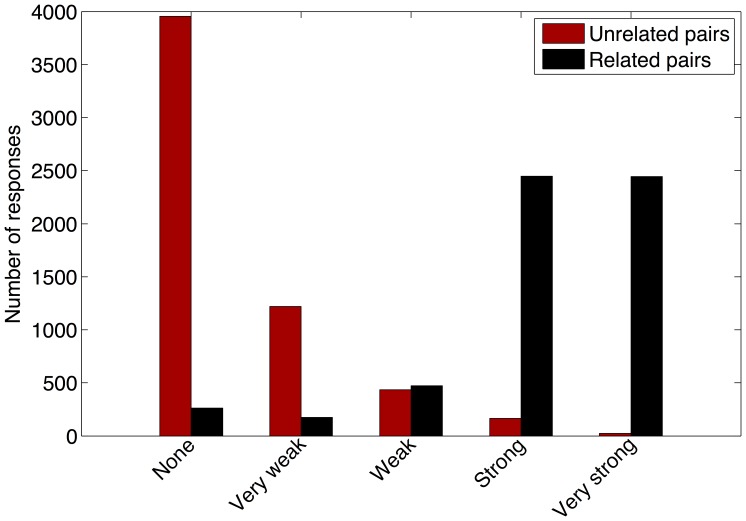
Histogram of perceived relation between word pairs of both sets. The 5-point scale on degree of relatedness is on the x-axis and the number of responses per pre-determined category, related (black) versus unrelated (red), is on the y-axis.

To validate the stimuli, a web survey was conducted in parallel with the EEG measurements, where subjects were asked to rate all word pairs on a 5-point relatedness scale from *not related* to *very strongly related*. 31 native Dutch subjects, 4 male, participated in the survey, aged between 17 and 61, with a mean of 24.4 (±9.9). Two subjects were rejected as outliers (more than 10% of the responses differed more than 3 standard deviations from the mean). The results of the survey can be found in Figure 1. Since the word pairs were selected to be either strongly related or not related at all, responses are predicted to be at the extremes of the scale. This is indeed the case, however there is some overlap in responses between the two sets. 13% of the responses do not correspond to the expected categorization. The unexpected categorization is not centered around a small amount of word pairs, but spread out over many, suggesting they are due to inter-subject variability in word knowledge and subjectivity in association rather than an error in the selection of the word pairs. 3% of the responses to unrelated pairs are labeled as related (strong relation and very strong relation), 7% of the responses to related pairs are labeled as unrelated (no relation and very weak relation). Another explanation for more related pairs being labeled as unrelated could be that, when subjects do not know the meaning of a word, they will label it as unrelated.

### Procedure

**Figure 2:**
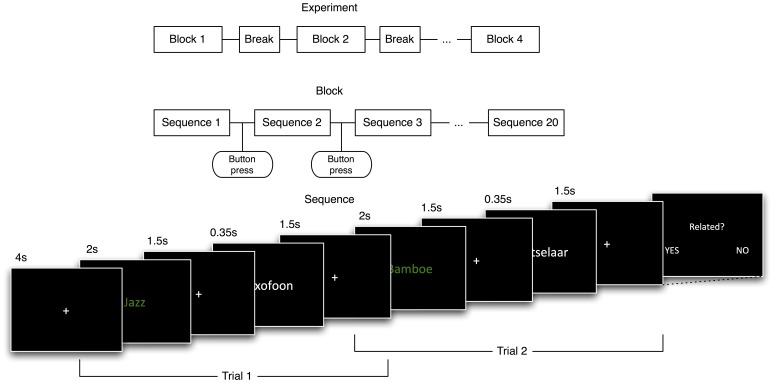
Schematic overview the experimental design. From global in time (top), to local in time (bottom).

Subjects were seated in a chair in front of a computer screen. After receiving the instructions, subjects first completed a short practice block in which they could familiarize with the task. The actual experiment is graphically represented in Figure 2. Subjects were presented with four blocks of about 15 minutes with a short pause between blocks. Each block consisted of twenty sequences, which in turn consisted of a baseline period of four seconds and five trials. One word pair was presented per trial. Subjects had to press a button to proceed from one sequence to the next. In each trial, first the prime was presented using a green colored font for 2000 ms. Next, a fixation cross appeared for 1500 ms, followed by the probe, presented in a white colored font. The probe was visible for 350 ms, followed by another fixation cross for 1500 ms. Subjects were instructed to pay attention to the words appearing on the screen and to determine whether the white probe-word was related to the green prime-word. To ensure subjects kept paying attention during the experiment, each block had 6 catch trials randomly distributed over the sequences. In a catch trial the subject was asked whether the last two words presented were related or not and they had to respond using two buttons. The word pair the subjects were asked about was always the last pair in a sequence.

### Equipment

The stimuli were presented with Psychtoolbox [20]–[22] version 3.0.8 running in Matlab 7.4. The stimuli were displayed on a 17" TFT screen, with a refresh rate of 60 Hz. The data was recorded using 64 sintered Ag/AgCl active electrodes using a Biosemi ActiveTwo AD-box and sampled at 2048 Hz. The electrodes were placed according to the 10/20 electrode system [23]. The EEG was recorded in an electrically shielded room. The EEG offset for each channel was kept below 25 *μ*V. A button box was used to allow participants to answer the catch trials and start the next sequence.

### Data Analysis

All preprocessing was done using the Fieldtrip toolbox [24]. Two different pipelines were used in data analysis. One for the grand average ERP statistics and one for the single-trial classification.

For the grand average ERPs the data was sliced to the trial level, i.e. from prime onset to second fixation cross offset with 0 at probe onset (−3.5 s–1.85 s). Next, the data was temporally down-sampled to 256 Hz. The data was detrended, a low-pass filter was applied at 30 Hz, and a linked-mastoid reference was computed. Relative baseline correction was applied using data from 100 ms before probe onset to probe onset. The preprocessing parameters were chosen to be able to compare them to other semantic priming experiments [3], [8], [10], [25]. To test for significant differences between the two conditions the cluster-based non-parametric statistic described by Maris and Oostenveld [26] was used. This test corrects for the multiple comparisons problem by incorporating a permutation test. For the statistical test the time of interest was set from 0 to 1000 ms after probe onset, and all 64 channels were used.

For the single-trial classification the data was again sliced to the trial level. It was detrended, bandpass filtered between 0.1 and 10 Hz and temporally down sampled to 32 Hz to reduce the number of features. Next, a linked-mastoid reference was computed. The time of interest was set from 0 to 1000 ms after probe onset, and all 64 channels were used, resulting in 2048 features (64 channels×32 time points). The preprocessing parameters were chosen to allow comparison with other classification analyses of single-trial ERPs [27]. Classification was performed using an *L*_2_ regularized logistic regression algorithm [11]. The regularization parameter (C) that was used resulted from a simple grid search where the variance in all the data is used as an estimate of the scale of the data, which is then multiplied by [.001.01.1 1 10 100]. This range has been shown to result in a high performance [27]. Two classification procedures were performed. First, the classifier was trained for each subject, ten-fold cross-validation was applied where each fold consisted of 360 training epochs and 40 test epochs. The data was divided into ten equally sized blocks of sequential trials, each block was designated as validation set in one of the folds. Second, to determine the generalizability of the signal used by the classifier, leave one subject out cross-validation was applied. This resulted in 4400 training epochs and 400 test epochs, where all the tests epochs belong to a single subject. A binomial statistical test was used to determine whether classification accuracies differed significantly from chance level (50%).

In order to be able to compare the classification results with other studies the Information Transfer Rate (ITR) is calculated. This measure combines the accuracy, the number of classes and the time needed for a classification. Wolpaw et al. [28] defined the ITR for a BCI as
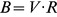
(1)where *B* is the ITR in bits per second, *V* is the number of classifications per second and *R* is the amount of information gained per classification, where R depends on the accuracy and the number of classes. For details, see Wolpaw et al. [28].

## Results

### Grand Average ERPs

**Figure 3:**
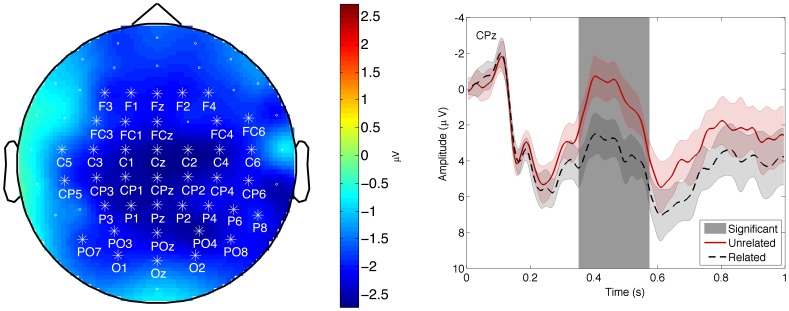
Grand average results for the negative component. Left panel: A topographic representation of the negative component between 330–600 ms. The marked channels show a significant difference between related and unrelated probe responses. Right panel: ERP waveforms for channel Cz for related (black, dashed) and unrelated (red, solid). The area around each line represents the standard deviation, corrected for a within subject design ([37], p. 361–366). Channel Cz has been chosen as an example channel, as other significant channels are similar. Areas marked in grey show a significant difference.

The grand average ERP responses to the two conditions (related and unrelated word pairs) were calculated for each channel and each time point. A cluster-based non-parametric statistic [26] was used to determine whether the difference between the two conditions was significant. The significance-level was set to 0.01. The statistic returned one significant cluster between 330 and 600 milliseconds after probe onset. This cluster is mostly located centrally on the scalp, see the left panel of Figure 3, channels with more than 100 ms of significant different time-points are indicated with an asterisk. A representative channel was selected from these channels; channel CPz, which is shown in the right panel of Figure 3. It shows an enhanced (more negative) N400 response for unrelated probes compared to related probes. This difference remains to the end of the trial. However, it is no longer statistically significant outside the N400 window.

### Single-Trial Detection

**Figure 4:**
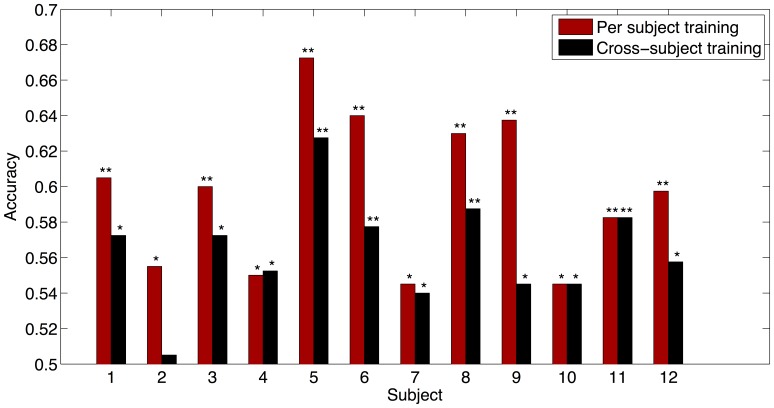
Classification accuracies for the individually trained classifier and the classifier trained across subjects. Accuracies are mean accuracies of test set performance over ten folds. (* 0.001<p<0.05, **p<0.001).

The results of the classification can be found in Figure 4. The accuracies for the classifier trained on individual subjects can be seen on the left and the accuracies for the classifier trained over subjects can be seen on the right. The reported accuracies are mean accuracies of test set performance over ten folds.

When calculating the ITRs using Equation (1) with the time required to gather the data needed to make a classification (5.35 s), the mean ITR is 0.36±0.29 (Maximum: 0.98) for the individually trained classifier and 0.16±0.14 (Maximum: 0.53) for the classifier trained over subjects.

## Discussion

The results show one cluster around CPz where the response to related word pairs differs significantly from the response to unrelated word pairs; a central negative cluster. This cluster shows the typical N400 effect found earlier in semantic priming studies [2]–[4], [8]–[10]. The late negative trend, has also been found in earlier studies [4], [8], [25]. The differences found in the responses between related and unrelated pairs are not caused by differences in word frequency, letter count or syllable counts, as the means were the same for both conditions for each of these possible confounds.

When training the classifier for each individual subject, the single-trial detection accuracies vary between 54% and 67%, where in all subjects the accuracy is significantly above chance level (50%). Even when training the classifier on data from other subjects, 11 out of 12 subjects show an accuracy significantly above chance level. This shows that the classifier is able to use a component in the subject’s response that is the same over all subjects, pointing to a general effect.

There are a number of other ERP components which have also been studied at the single trial level: mainly the P300, Mismatch Negativity (MMN), and Error-Related Potential (ErrP). The P300 ERP can be divided into four conditions: (i) the overt visual P300, which has a detection accuracy of 77–85% [14], [29]–[31], (ii) the covert visual P300, which has a detection accuracy of around 58% [31], (iii) the tactile P300, with a detection accuracy of around 67% [31], and (iv) the auditory P300, with a detection accuracy of 65–74% [32], [33]. The overt P300 results are higher than the other condtions, because there the subject foveates on the intended stimulus, leading to differences in the primary visual responses, which are also included in the classification, which means it is not detection of only the P300 component. The Mismatch Negativity has been detected with an accuracy of 69% [34], and the Error-Related Potential with an accuracy between 66–80% [35], [36].

It has been established that the amplitude of the N400 response is correlated with the degree of relatedness between the prime and probe [16]. In the current experiment the stimuli have been selected in such a way that the two categories the classifier needs to distinguish are as far apart as possible, i.e., the mean difference in relatedness of prime and probe is as large as possible. In a practical setting where such a constraint is not possible, we expect the detection accuracy to drop slightly, as the difference in amplitude of the N400 will be smaller in the situation where prime and probe are less strongly related. In future work, we will look at the effect of a lower degree of relatedness on the classification performance.

The significant classification results for the cross-subject classifier would allow the detection of semantic priming from the start of an experiment. Generally when using an online classifier it needs to be trained first. This is done by gathering data where one knows to which class each data segment belongs, i.e., a training block. A training block usually takes about ten to twenty minutes. However, when the classifier can be trained on data from previous subjects, new subjects can skip the training block. The classifier could later improve, i.e., adapt to an individual user, by retraining when subject data becomes available. However, the lower classification accuracy would mean that the performance is worse than when including a training block.

The ITRs achieved here are low compared to other word communication BCIs, such as the visual speller [13]. However, by relying only on the users’ ability to identify associated concepts this approach offers the potential to detect a desired concept without the user having to know the correct word or even how spell it. This offers potential applications beyond simple communication, such as helping aphasic’s communicate the concept they are unable to say, or to help other users stuck in a ‘tip-of-the-tongue’ state.

Concluding, it is possible to detect semantic priming at the single-trial level, though the classification accuracies are low. The classification over subjects shows that there is a common response that is the same in all subjects and this response can be exploited for the detection of semantic priming.

When using the semantic priming response for BCI purposes using the timing parameters described here, it takes 5.35 seconds to present one probe. This could be reduced by using the timing parameters described by Brown and Hagoort [8], reducing the time per probe to 3.94 seconds. Both these methods show one probe per target. If we show multiple probes for one target we could bring the time per probe down to about 1.5 seconds. This would increase the Information Transfer Rates reported in the results section. The ITR would increase from 0.36±0.29 (Best: 0.98) to 1.3±1.0 (Best: 3.5) for the individually trained classifier and from 0.16±0.14 (Best: 0.53) to 0.57±0.50 (Best: 1.9) for the classifier trained over subjects.

We have shown that it is possible to detect semantic priming at the single-trial level and that the single-trial accuracies differ significantly from chance level for all measured participants.

## Supporting Information

Stimuli S1 Full list of stimuli. List of all stimuli used in the experiment, including the information extracted from Celex.
(PDF)
