# Supplementary material for: Detecting Semantic Priming at the Single-Trial Level
Source: PLoS One. 2013 Apr 2;8(4):e60377. doi: 10.1371/journal.pone.0060377 (PMC3615017; doi:10.1371/journal.pone.0060377)
Supplement: Stimuli S1 — List of all stimuli used in the experiment, including the information extracted from Celex. (PDF) [file pone.0060377.s001.pdf]

Prime				Probe			
Word	LogFreq	LetCnt	SylCnt	Word	LogFreq	LetCnt	SylCnt
jazz	0.301	4	1	saxofoon	0	8	2
wasknijper	0	10	3	was	0	3	1
bestek	0.9542	6	2	vork	1.0792	4	1
krik	0	4	1	pech	0.8451	4	1
eiwit	0.9542	5	2	eigeel	0.301	6	2
bezem	0.6021	5	2	heks	0	4	1
dam	0.699	3	1	bever	0	5	2
spinazie	0.4771	8	3	groen	0	5	1
tennisracket	0	12	4	tennisbal	0	9	3
pompelmoes	0	10	3	bitter	0	6	2
walrus	0	6	2	snor	0	4	1
racket	0	6	2	tennis	0.301	6	2
confituur	0	9	3	aardbei	0.699	7	2
periscoop	0	9	3	duikboot	0.301	8	2
vlot	0.4771	4	1	snel	0	4	1
puck	0	4	1	hockey	0	6	2
beroep	2.0453	6	2	werk	2.7566	4	1
volleybal	0	9	3	net	1.2553	3	1
zoogdier	0.7782	8	2	mens	0	4	1
libel	0	5	2	vijver	1.1761	6	2
naald	1.2041	5	1	draad	1.4472	5	1
spek	0	4	1	varken	1.3617	6	2
mier	0.8451	4	1	klein	0.301	5	1
tol	0.699	3	1	speelgoed	1.1461	9	2
pak	1.7853	3	1	kostuum	1.0792	7	2
vet	1.1761	3	1	dik	0	3	1
inbraak	0.6021	7	2	dief	1.1139	4	1
zwaard	1.1761	6	1	ridder	1.1139	6	2
aambeeld	0	8	2	smid	0.7782	4	1
puzzel	0.6021	6	2	stukje	1.9494	6	2
zakdoek	1.3222	7	2	snuit	0.7782	5	1
ton	1.4771	3	1	vat	0.699	3	1
verjaardag	1.3222	10	3	cadeau	1.3424	6	2
vampier	0.6021	7	2	bloed	0	5	1
roofvogel	0.301	9	3	arend	0.6021	5	2
gewei	0	5	2	hert	0.8451	4	1
denneappel	0	10	4	bos	0.301	3	1
web	0.6021	3	1	spin	0.9542	4	1
balkon	1.2304	6	2	terras	1.6128	6	2
rups	0	4	1	vlinder	1	7	2
kapstok	0.699	7	2	jas	0	3	1
moer	0.4771	4	1	vijs	0	4	1
hazelnoot	0	9	3	eekhoorn	0.4771	8	2
wei	1.0414	3	1	koe	1.5563	3	1
punt	1.2553	4	1	komma	0.4771	5	2
tram	1.301	4	1	spoor	0.4771	5	1
tweeloop	0	8	2	geweer	1.6628	6	2
riek	0	4	1	hooi	0.8451	4	1
artisjok	0	8	3	groente	1.3617	7	2
notenbalk	0	9	3	muziek	2.0607	6	2

TABLE S1. Related I

Prime				Probe			
Word	LogFreq	LetCnt	SylCnt	Word	LogFreq	LetCnt	SylCnt
mei	1.7924	3	1	lente	1.2788	5	2
bruid	1.0414	5	1	bruidegom	0.699	9	3
dolfijn	0.6021	7	2	flipper	0	7	2
goal	0.301	4	1	voetbal	0	7	2
slip	0.6021	4	1	ondergoed	0.9542	9	3
harpoen	0	7	2	walvis	0.4771	6	2
aas	0.301	3	1	worm	1	4	1
clementine	0	10	4	mandarijn	0.4771	9	3
wittekool	0	9	3	rodekool	0	8	3
prehistorie	0	11	4	oermens	0	7	2
tekenfilm	0	9	3	cartoon	0	7	2
ijshockey	0	9	3	schaats	0	7	1
pasta	0.4771	5	2	spaghetti	0.4771	9	3
ooievaar	0.301	8	3	nest	1.3802	4	1
kuif	0.4771	4	1	gel	0	3	1
kurk	0	4	1	fles	2.0492	4	1
masker	1.2553	6	2	carnaval	0.4771	8	3
raam	2.2405	4	1	venster	1.4624	7	2
rasp	0	4	1	wortel	1.5682	6	2
zilver	1.0792	6	2	juweel	1.1139	6	2
muizeval	0	8	3	muis	1.3222	4	1
eiland	1.8921	6	2	palmboom	0.4771	8	2
molen	1.0414	5	2	wiek	0.4771	4	1
stekker	0.301	7	2	stopcontact	0.301	11	3
neushoorn	0	9	2	grijs	0.699	5	1
giraf	0	5	2	vlek	0	4	1
theepot	0.301	7	2	thee	0	4	1
kalkoen	0.4771	7	2	kerstmis	0	8	2
strijkplank	0	11	2	strijkijzer	0	11	3
rugbybal	0	8	3	ovaal	0	5	2
leguaan	0	7	3	reptiel	0.4771	7	2
badminton	0	9	3	pluimpje	0	8	2
ochtend	1.9031	7	2	moe	0.6021	3	1
inktvis	0.301	7	2	tentakel	0	8	3
fanfare	0.4771	7	3	trompet	0.6021	7	2
jungle	0.699	6	2	oerwoud	1	7	2
triangel	0	8	3	driehoek	0.9542	8	2
pijp	1.3979	4	1	tabak	1.1139	5	2
doedelzak	0	9	3	schot	0.4771	5	1
kameel	0.8451	6	2	bult	0.6021	4	1
slurf	0	5	1	olifant	1	7	3
klok	1.5682	4	1	wijzer	0.6021	6	2
ezel	1.0792	4	2	dom	0.301	3	1
wandelstok	0.699	10	3	oud	0.4771	3	1
brand	1.6532	5	1	brandweer	0.6021	9	2
goudvis	0.301	7	2	bokaal	0.301	6	2
vlaai	0	5	1	taart	1	5	1
bat	0	3	1	honkbal	0	7	2
ansjovis	0.301	8	3	pizza	0	5	2
ijsje	0.4771	5	2	vanille	0.301	7	3

Table S2. Related II

Prime				Probe			
Word	LogFreq	LetCnt	SylCnt	Word	LogFreq	LetCnt	SylCnt
boormachine	0	11	4	lawaai	1.4914	6	2
vuilbak	0	7	2	afval	1.1139	5	2
engel	1.415	5	2	hemel	0.301	5	2
orkest	1.0414	6	2	dirigent	0.8451	8	3
dorst	1.1461	5	1	drinken	1.699	7	2
kaaiman	0	7	2	krokodil	0.699	8	3
krokus	0	6	2	vakantie	1.7404	8	3
wafel	0.301	5	2	slagroom	0.6021	8	2
luier	0.8451	5	2	baby	1.8976	4	2
noordpool	0.301	9	2	zuidpool	0.301	8	2
gebak	0.6021	5	2	cake	0.4771	4	1
loodgieter	0.301	10	3	buis	0.9031	4	1
sinterklaas	0.6021	11	3	zwartepiet	0	10	3
kerstman	0	8	2	kerstboom	0.699	9	2
ballet	0.6021	6	2	roze	0.301	4	2
stewardess	0.4771	10	3	vliegtuig	1.716	9	2
puree	0.301	5	2	aardappel	1.4472	9	3
kwark	0.301	5	1	yoghurt	0.4771	7	2
geur	1.8451	4	1	parfum	1.1761	6	2
amfibie	0	7	3	kikker	0.9542	6	2
ring	1.5315	4	1	trouw	0	5	1
beha	0.699	4	2	borst	0	5	1
statief	0	7	2	fototoestel	0.6021	11	4
scharnier	0.4771	9	2	piep	0	4	1
tandarts	1.1139	8	2	pijn	2.1847	4	1
tandenborstel	0.6021	13	4	tandpasta	0.4771	9	3
boks	0	4	1	handschoen	1.1139	10	2
sinaasappel	0.9031	11	4	oranje	0.7782	6	3
gereedschap	1.0414	11	3	hamer	1.0414	5	2
keu	0	3	1	biljart	0	7	2
schaar	0	6	1	knip	0	4	1
skelet	0.699	6	2	geraamte	0.699	8	3
druk	1.9868	4	1	stress	1.0792	6	1
kruk	0.9542	4	1	bar	0	3	1
rolschaats	0	10	2	val	0	3	1
muur	0	4	1	baksteen	0.699	8	2
keukengerief	0	12	4	lepel	1.2553	5	2
fabriek	1.6435	7	2	arbeider	1.7853	8	3
tankstation	0	11	4	benzine	0.9542	7	3
zondag	1.6128	6	2	weekend	1.2304	7	2
kievit	0.301	6	2	vogel	1.9823	5	2
fagot	0	5	2	instrument	1.6812	10	3
kok	0	3	1	muts	0.8451	4	1
chips	0.6021	5	1	paprika	0.699	7	3
autosnelweg	0	11	4	file	0.4771	4	1
assepoester	0	11	4	sprookje	1.2041	8	2
badkamer	1.3802	8	3	bad	1.3802	3	1
lava	0.301	4	2	vulkaan	0.7782	7	2
aubergine	0.301	9	3	paars	0.4771	5	1
zoet	0	4	1	snoep	0.301	5	1

Table S3. Related III

Prime				Probe			
Word	LogFreq	LetCnt	SylCnt	Word	LogFreq	LetCnt	SylCnt
ruw	0	3	1	schuurpapier	0	12	3
kano	0.699	4	2	varen	0.4771	5	2
metaal	0	6	2	ijzer	1.2553	5	2
drank	1.5911	5	1	cola	0.699	4	2
vijl	0	4	1	nagel	1.3424	5	2
oliebol	0	7	3	kermis	1.0414	6	2
pauw	0.7782	4	1	veer	1.1461	4	1
waterput	0.301	8	3	emmer	1.3424	5	2
camping	0.4771	7	2	caravan	0.6021	7	3
brievenbus	0.7782	10	3	post	1	4	1
vrede	1.716	5	2	duif	0	4	1
croissant	0.301	9	2	ontbijt	1.4314	7	2
rechtbank	1.3802	9	2	advocaat	1.4914	8	3
magie	1.0414	5	2	tovenaar	0.8451	8	3
bok	0	3	1	geit	0.301	4	1
judo	0	4	2	mat	0.8451	3	1
vijg	0	4	1	plat	0	4	1
plan	2.3032	4	1	idee	0.8451	4	2
dinosaurus	0	10	4	groot	0	5	1
pull	0	4	1	trui	0	4	1
egel	0.4771	4	2	stekel	0.4771	6	2
hak	0	3	1	schoen	1.8325	6	1
hark	0.301	4	1	tuin	2.0755	4	1
paperclip	0	9	3	papier	2.0531	6	2
wol	1	3	1	schaap	1.415	6	1
bed	2.4771	3	1	slaap	0.4771	5	1
rad	0.9031	3	1	fortuin	0.9031	7	2
kleerkast	0.6021	9	2	kleren	1.6335	6	2
boete	0.9542	5	2	politie	1.9777	7	3
boter	1.3617	5	2	boterham	1.2553	8	3
thermometer	0.4771	11	4	koorts	1.3424	6	1
paddestoel	0.9542	10	3	kabouter	0.699	8	3
chocolade	0.699	9	4	bruin	0	5	1
snaar	0.699	5	1	gitaar	0.7782	6	2
synthesizer	0	11	4	piano	1.2041	5	3
brandweerwagen	0	14	4	sirene	1	6	3
palet	0	5	2	schilder	1.4472	8	2
moto	0	4	2	snelheid	1.6021	8	2
apotheker	0.9031	9	4	medicijn	1.415	8	3
cel	1.6628	3	1	gevangenis	1.6335	10	4
deksel	1.2553	6	2	pot	0.699	3	1
winkelbediende	0	14	5	kassa	0.8451	5	2
toneel	1.5441	6	2	acteur	1.2553	6	2
safari	0	6	3	jeep	1	4	1
weegschaal	0.699	10	2	gewicht	1.6232	7	2
oudheid	1.1139	7	2	geschiedenis	2.1367	12	4
angel	0.699	5	2	wesp	0.6021	4	1
mango	0	5	2	fruit	1.1139	5	1
veiligheidsspeld	0	16	4	prik	0.4771	4	1
circus	0.8451	6	2	clown	0.699	5	1

Table S4. Related IV

Prime				Probe			
Word	LogFreq	LetCnt	SylCnt	Word	LogFreq	LetCnt	SylCnt
vink	0.301	4	1	turkoois	0	8	2
hovercraft	0	10	3	los	0	3	1
rugzak	0.9031	6	2	ford	1	4	1
kort	0	4	1	lens	0.7782	4	1
dieet	1.0414	5	2	mimiek	0.301	6	2
merel	0.699	5	2	flip	0	4	1
dolk	0.699	4	1	lager	0	5	2
spinneweb	0.4771	9	3	bruis	0	5	1
tafeltennis	0	11	4	geknabbel	0	9	3
stekelbaars	0	11	3	gebalk	0	6	2
sweater	0	7	2	vaak	0	4	1
hamster	0	7	2	antiek	0.301	6	2
herbivoor	0	9	3	wroeging	0.699	8	2
kokosnoot	0	9	3	huifkar	0.301	7	2
jacht	0.4771	5	1	fret	0	4	1
rog	0	3	1	maaier	0	6	2
gevaar	2.0645	6	2	zaak	2.6274	4	1
supporter	0	9	3	wolf	1.2304	4	1
knuppel	0.7782	7	2	smul	0	4	1
eland	0	5	2	eerbied	1.2041	7	2
haan	1.2304	4	1	laars	1.4314	5	1
meer	0	4	1	verkoop	1.3222	7	2
koek	0.9542	4	1	sloop	0.301	5	1
tang	0.699	4	1	opbrengst	1.2041	9	2
berg	1.7404	4	1	drankje	1.0414	7	2
tank	1.2304	4	1	kul	0	3	1
cocktail	0.6021	8	2	vonk	1.0414	4	1
kraan	1.1461	5	1	kachel	1.1761	6	2
knuffel	0	7	2	hasj	0.699	4	1
handtas	0.6021	7	2	morgen	2.0334	6	2
mantel	1.2553	6	2	zwijn	0.7782	5	1
blok	1.5441	4	1	juf	0.699	3	1
vrachtwagen	1.301	11	3	drukte	1.2553	6	2
magneet	0.4771	7	2	plint	0	5	1
hamburger	0.301	9	3	sage	0.699	4	2
lychee	0	6	2	roem	0.9542	4	1
madeliefje	0	10	4	pub	0.4771	3	1
prei	0.6021	4	1	krul	1.0792	4	1
piloot	1.2041	6	2	planeet	1.4914	7	2
pit	0	3	1	ontzag	1	6	2
haring	0.699	6	2	mars	0	4	1
kan	0.4771	3	1	sjiek	0	5	1
breekijzer	0	10	3	geschut	0.4771	7	2
sap	1.0414	3	1	cent	1.415	4	1
kar	1.2041	3	1	galop	0.6021	5	2
hoorn	1.3424	5	1	junk	0.4771	4	1
eenhoorn	0	8	2	vertrek	1.8062	7	2
poef	0	4	1	box	0.699	3	1
bazooka	0	7	3	lengte	1.4314	6	2
rabarber	0	8	3	begrip	2.2201	6	2

Table S5. Unrelated I

Prime				Probe			
Word	LogFreq	LetCnt	SylCnt	Word	LogFreq	LetCnt	SylCnt
golf	1.7853	4	1	leugen	1.3979	6	2
zwaan	0.9031	5	1	levering	0.699	8	3
zwaluw	0.699	6	2	draagtas	0	8	2
biet	0.4771	4	1	spikkel	0	7	2
gesp	0.4771	4	1	reflectie	0.7782	9	3
aalbes	0	6	2	extreem	0.301	7	2
harp	0.301	4	1	goot	0.7782	4	1
dromedaris	0	10	4	visserij	0.699	8	3
luchtballon	0	11	3	decadent	0	8	3
accordeon	0	9	4	turnles	0	7	2
verfborstel	0	11	3	snotaap	0	7	2
microgolf	0	9	3	blaas	0	5	1
bamboe	0.301	6	2	metselaar	0.4771	9	3
elastiek	0.4771	8	3	gek	1.3617	3	1
ros	0.301	3	1	dol	0	3	1
slee	0	4	1	strijd	2.0828	6	1
matroos	1.1461	7	2	verliezer	0.6021	9	3
hart	2.2788	4	1	thema	1.4914	5	2
roest	0	5	1	procent	1.7709	7	2
beker	1.1761	5	2	versie	1.3222	6	2
basketbal	0	9	3	stier	1.1139	5	1
natuur	1.9685	6	2	cipier	0.4771	6	2
anker	0.9542	5	2	claxon	0.4771	6	1
pudding	0.4771	7	2	schemerlamp	0.4771	11	3
luipaard	0	8	2	speech	0.4771	6	1
koevoet	0	7	2	aal	0	3	1
topje	0.301	5	2	stamp	0	5	1
kever	0.4771	5	2	fietsband	0	9	2
stinkdier	0	9	2	grootbedrijf	0	12	3
appelflap	0	9	3	duo	0	3	2
hazelworm	0	9	3	duiker	0.4771	6	2
vingerhoed	0	10	3	bloedgroep	0	10	2
geweld	1.7559	6	2	zaag	0.4771	4	1
fazant	0.4771	6	2	regenpijp	0	9	3
kanarie	0.4771	7	3	klavier	0.301	7	2
kampvuur	0.6021	8	2	schotel	1.1139	7	2
plamuurmes	0	10	3	eetzaal	0.8451	7	2
lamp	1.4914	4	1	serie	1.415	5	2
pistolet	0	8	3	staaf	0.7782	5	1
tijger	0.8451	6	2	lei	0.301	3	1
vest	0	4	1	uitstapje	0.8451	9	3
struik	1.4771	6	1	hefboom	0.4771	7	2
ober	1.1461	4	2	buil	0	4	1
postzegel	0.699	9	3	truck	0.301	5	1
pad	1.7709	3	1	waakhond	0.4771	8	2
oorbel	0.301	6	2	knielap	0	7	2
squash	0	6	1	cape	0.699	4	1
kruis	0	5	1	frisdrank	0	9	2
rijbewijs	0.4771	9	3	kookwas	0	7	2
toeter	0.301	6	2	foltering	0.301	9	3

TABLE S6. Unrelated II

Prime				Probe			
Word	LogFreq	LetCnt	SylCnt	Word	LogFreq	LetCnt	SylCnt
onderlegger	0	11	4	leraar	1.8633	6	2
blokfluit	0	9	2	ego	1.1761	3	2
akker	1.2041	5	2	zitvlak	0.301	7	2
training	1.2553	8	2	prestige	0.8451	8	3
bijl	1.0414	4	1	herstel	1.3617	7	2
oorworm	0	7	2	beschutting	0.699	11	3
klaproos	0	8	2	werkgever	1.4771	9	3
bizon	0	5	2	software	0.4771	8	2
hobby	0.7782	5	2	voedsel	1.8195	7	2
roomsoes	0	8	2	doodskop	0.301	8	2
toga	0.301	4	2	shot	0.4771	4	1
bloedzuiger	0	11	3	berm	0.8451	4	1
kruiwagen	0.699	9	3	versiersel	0.301	10	3
glijbaan	0	8	2	sprinkhaan	0.301	10	2
deurknop	0.6021	8	2	piste	0	5	2
wasmachine	0.4771	10	4	landschap	1.6721	9	2
schommel	0.301	8	2	grootvader	1.5315	10	3
snoek	0.301	5	1	autobus	0.4771	7	3
vuist	1.5682	5	1	toerist	1.3424	7	2
contrabas	0	9	3	wolkje	0.6021	6	2
jurk	1.6232	4	1	timer	0	5	2
pony	0.6021	4	2	ijzel	0	5	2
plastiek	0	8	2	verspilling	0.6021	11	3
bromfiets	0.4771	9	2	papje	0	5	2
vloeistof	1.1139	9	2	orde	2.2014	4	2
verrekijker	0.7782	11	4	beroemdheid	0.6021	11	3
step	0	4	1	verwijzing	1.2553	10	3
portefeuille	1.0414	12	4	nadenken	1.0792	8	3
revolver	1.1761	8	3	jurist	0.9542	6	2
orka	0	4	2	aardas	0	6	2
slijper	0	7	2	broos	0	5	1
fornuis	0.699	7	2	isolatie	0.6021	8	4
lijn	2.017	4	1	rund	0.699	4	1
kanon	1.0414	5	2	balg	0	4	1
zwempak	0	7	2	zweef	0	5	1
schaaf	0	6	1	loper	0.699	5	2
platenspeler	0	12	4	chip	1.0792	4	1
schrijven	1.8865	9	2	eigenschap	1.7993	10	3
opvoedster	0	10	3	utopie	0.7782	6	3
vleugel	1.5798	7	2	eindpunt	0.7782	8	2
beschuit	0.301	8	2	toekomst	2.1173	8	2
iglo	0	4	2	politicus	1.5315	9	4
rock	0.301	4	1	dun	0.6021	3	1
specht	0.301	6	1	verpleger	0.6021	9	3
klavecimbel	0	11	4	schare	0.4771	6	2
tomatensaus	0	11	4	dienblad	0.699	8	2
psycholoog	1.6128	10	3	vocht	1.2041	5	1
gekko	0	5	2	aardbol	0.4771	7	2
zeehond	0.301	7	2	krot	0.4771	4	1
potvis	0	6	2	bout	0.301	4	1

TABLE S7. Unrelated III

Prime				Probe			
Word	LogFreq	LetCnt	SylCnt	Word	LogFreq	LetCnt	SylCnt
banjo	0	5	2	verkwikking	0	11	3
paling	0.7782	6	2	geldstuk	0.301	8	2
platvis	0	7	2	cabine	1.0414	6	3
poot	1.6021	4	1	spuit	0.4771	5	1
mot	0.301	3	1	dollar	1.6335	6	2
trombone	0	8	3	commando	1.0414	8	3
gif	0.699	3	1	zusje	1.3617	5	2
draaimolen	0.301	10	3	rondje	0.9031	6	2
forel	0.4771	5	2	agenda	1.0414	6	3
pantoffel	0.699	9	3	robot	0.7782	5	2
knie	1.9243	4	1	rit	0	3	1
trechter	0.301	8	2	tegenzin	1.1761	8	3
priester	1.6628	8	2	voldoening	1.2304	10	3
vlieger	0.7782	7	2	casino	1.1139	6	3
micro	0	5	2	teddy	0.301	5	2
lolly	0	5	2	album	0.6021	5	2
ajuin	0	5	2	krukas	0	6	2
geluid	2.1644	6	2	made	0.6021	4	2
piranha	0	7	3	drum	0	4	1
stop	0.301	4	1	dadel	0	5	2
kiwi	0.301	4	2	oogvocht	0	8	2
wok	0.301	3	1	leiding	1.8573	7	2
hoef	0.301	4	1	produkt	1.9345	7	2
basgitaar	0	9	3	stadium	1.6532	7	3
mus	1.2553	3	1	contract	1.3222	8	2
kant	2.4639	4	1	slaapzak	0.6021	8	2
vlo	0.6021	3	1	hoofdpijn	1.301	9	2
sandwich	0.4771	8	2	instituut	1.4624	9	3
zalm	0.699	4	1	ambtenaar	1.7634	9	3
worst	1.0414	5	1	opluchting	1.2553	10	3
kinderwagen	0.6021	11	4	vracht	0.699	6	1
hagedis	0.7782	7	3	kleinkind	0.9031	9	2
aquarium	0.699	8	4	roeispaan	0	9	2
uil	0.9031	3	1	omhulsel	0.4771	8	3
marktkramer	0	11	3	tasje	0.8451	5	2
schommelstoel	0.301	13	3	symfonie	0.699	8	3
krekel	0.4771	6	2	schoonheid	1.6435	10	2
koala	0	5	3	vernieuwing	1.415	11	3
viooltje	0.4771	8	3	bestemming	1.3424	10	3
gordijn	1.6532	7	2	geneesmiddel	1.3979	12	4
viool	1.0792	5	2	kauw	0	4	1
cassetterecorder	0	16	6	opvoeder	0.8451	8	3
koningin	1.6128	8	3	herhaling	1.2788	9	3
kameleon	0	8	4	dia	0.6021	3	2
ekster	0.4771	6	2	tijdschrift	1.5911	11	2
fluit	0.699	5	1	psychologie	1.7993	11	4
wekker	0.8451	6	2	sabbat	0	6	2
perzik	0	6	2	bont	0.301	4	1
verkeerslicht	0.301	13	3	kers	0	4	1
tomaat	0.9542	6	2	nectar	0	6	2

Table S8. Unrelated IV
